# Diet segregation in American bison (*Bison bison*) of Yellowstone National Park (Wyoming, USA)

**DOI:** 10.1186/s12898-017-0137-9

**Published:** 2017-07-14

**Authors:** John L. Berini, Catherine Badgley

**Affiliations:** 1 Department of Fisheries, Wildlife, and Conservation Biology, University of Minnesota, 135 B Skok Hall, 2003 Upper Buford Circle, St. Paul, MN 55108-1052 USA; 2 Department of Ecology and Evolutionary Biology, University of Michigan, 1109 Geddes Avenue, Ann Arbor, MI 48109-1079 USA

## Abstract

**Background:**

Body size is a major factor in the nutritional ecology of ruminant mammals. Females, due to their smaller size and smaller rumen, have more rapid food-passage times than males and thereby require higher quality forage. Males are more efficient at converting high-fiber forage into usable energy and thus, are more concerned with quantity. American bison are sexually dimorphic and sexually segregate for the majority of their adult lives, and in Yellowstone National Park, they occur in two distinct subpopulations within the Northern and Central ranges. We used fecal nitrogen and stable isotopes of carbon and nitrogen from American bison to investigate sex-specific differences in diet composition, diet quality, and dietary breadth between the mating season and a time period spanning multiple years, and compared diet indicators for these different time periods between the Northern and Central ranges.

**Results:**

During mating season, diet composition of male and female American bison differed significantly; females had higher quality diets, and males had greater dietary breadth. Over the multi-year period, females had higher quality diets and males, greater dietary breadth. Diet segregation for bison in the Central Range was more pronounced during the mating season than for the multi-year period and females had higher quality diets than males. Finally, diet segregation in the Northern Range was more pronounced during the multi-year period than during the mating season, and males had greater dietary breadth.

**Conclusions:**

Female bison in Yellowstone National Park have higher quality diets than males, whereas males ingest a greater diversity of plants or plants parts, and bison from different ranges exhibited more pronounced diet segregation during different times. Collectively, our results suggest that diet segregation in bison of Yellowstone National Park is associated with sex-specific differences in nutritional demands. Altogether, our results highlight the importance of accounting for spatial and temporal heterogeneity when conducting dietary studies on wild ungulates.

## Background

Body size is a major factor in the nutritional ecology of ruminant mammals, as mass-specific energy demands generally decrease with increasing body mass [1]. On average, the ability of ungulates to convert high-fiber, low-quality forage into usable energy increases with body size, a trend known as the Jarman–Bell principle [2, 3]. Although the Jarman–Bell principle was first used to explain dietary differences among African ruminants of varying sizes [2, 3], evidence suggests that the principles upon which the Jarman–Bell principle is founded hold true for dietary differences within species as well, and therefore, may help explain sexual segregation in sexually dimorphic ruminants [4]. Applying the Jarman–Bell principle to size-dimorphic ruminants suggests that smaller individuals should be more selective feeders and have higher quality diets than larger individuals [2–4]. In sexual segregation theory, the forage-selection hypothesis suggests that females, due to their smaller size and smaller rumen, have more rapid food-passage times than males and thereby require higher quality forage in order to maintain body weight. Males, with larger size and longer food-retention times, are more efficient at converting high-fiber forage into usable energy and thus, are more concerned with quantity [5–7]. Although numerous hypotheses have been invoked to explain sexual segregation in ruminants (for a review see [8]), this phenomenon is best characterized as the differential use of space, habitat, or food by males and females.

Sexual dimorphism and segregation in large mammals are typically associated with sex-specific differences in energetic requirements and digestion [9]. For example, the activity budget hypothesis assumes that females are less efficient at digesting forage than males, and therefore spend more time foraging, whereas males spend more time ruminating [10]. While the forage-selection hypothesis also assumes that females are less efficient at digesting forage, this hypothesis predicts group formation based on dietary preferences as opposed to activity budget. American bison (*Bison bison*) are highly size-dimorphic, with the average male (800 kg) weighing roughly 350 kg more than the average female (450 kg; [11]). Reproductively active females have higher energy demands than males due to gestation and lactation, with energetic requirements peaking during early to mid-summer [12, 13]. The energetic demands of males also peak during early to mid-summer, when mature individuals are replenishing energy stores in preparation for the rut, which occurs during late summer [12, 14]. During the rut, male and female bison spend approximately 1 month in large mating groups, with males tending to potential mates in an effort to maximize breeding potential [15]. Throughout the rest of the year, males and females remain segregated. Collectively, the anatomical, behavioral, and physiological differences between male and female American bison make them an ideal species for studies of diet segregation. Previous studies of sexual segregation in American bison provide evidence for both the forage-selection hypothesis and the predation-risk hypothesis. The latter assumes that females and their calves are more vulnerable to predation than males and therefore select habitats that provide increased safety over increased forage quality [5, 16].

**Fig. 1:**
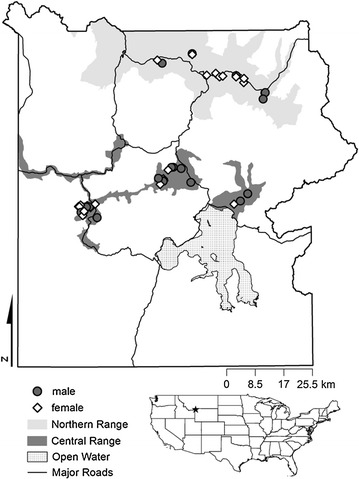
Distribution of carcass samples throughout the Northern and Central ranges in Yellowstone National Park (YNP). *Inset map* shows the location of YNP within the continental United States, as represented by the *star*

In Yellowstone National Park (YNP), the bison population consists of two geographically and ecologically distinct subpopulations. In the Northern and Central ranges of YNP (Fig. 1), bison populations differ in fetal growth rates [17], median calving dates [17], and tooth-wear patterns [18]. Differences in mtDNA haplotype frequencies between ranges provide evidence for assortative breeding [19], and since their reintroduction in 1995, wolf predation rates on bison have been higher in the Central Range than in the Northern Range [20, 21]. Additionally, higher altitude and colder temperatures in the Central Range result in deeper snow cover, which remains significantly longer than in the Northern Range [22, 23]. The Central Range also has higher levels of geothermal activity than the Northern Range, resulting in greater abundance of winter forage [24]. However, the quality of forage located on geothermal patches tends to be of relatively low quality, with elevated levels of fluoride and silica [25]. The two ranges also differ in bedrock geology and soil type [23]. The Northern Range is dominated by andesitic bedrock, which generates soils that are richer in plant-available nutrients and organic carbon compared to soils generated by rhyolitic bedrock, which is prevalent across the Central Range [23]. Population growth of bison in YNP is thought to be contingent on herbivore density and winter-forage availability [26]. In general, Yellowstone National Park is considered to be forage-limited [26], and population densities of both elk and bison are greater on the Northern Range compared to those of the Central Range [27, 28]. Spatial and temporal variations in the biotic and abiotic environments should be reflected in the quality, abundance, and distribution of forage available to bison throughout YNP and therefore, also reflected in the composition and quality of their diets.

If different sexes face different nutritional demands, then differences in forage quality and density-dependent influences on forage availability and selection should be reflected in how similar (or different) the diets of males and females are within each range. Moreover, the degree of diet segregation between males and females within each range should vary between the mating and non-mating seasons. In the Central Range, bison and elk densities are relatively low throughout the year [27, 28]. As a result, animals in this region of the park likely experience strong density-dependent effects during late summer, when animals form large, mixed-sex mating groups. Thus, diet segregation in the Central Range should be most pronounced during this time, and should be in sharp contrast to the degree of diet segregation we might observe throughout the rest of the year. In the Northern Range, bison and elk densities are considerably higher, and as a result, animals in this region of the park likely experience strong density-dependent effects throughout the entire year. While ungulate densities in the Northern Range may also peak during the mating season, differences in the degree of diet segregation between the mating and non-mating seasons are likely less pronounced than that which we might observe on the Central Range.

Using fecal nitrogen and stable isotopes of carbon (δ^13^C) and nitrogen (δ^15^N), we evaluated whether male and female bison in YNP exhibit diet segregation and whether the degree of diet segregation varies within each range during mating season and across a time period spanning multiple years. To determine whether diet segregation of bison in YNP is associated with sex-specific nutritional demands and whether these demands are met differently in each range, we tested four hypotheses. (1) Male and female bison have distinct diet compositions during the mating season and (2) across multiple years. (3) Male and female bison from the Central Range differ more in diet composition (i.e., show more pronounced diet segregation) during the mating season than across multiple years. (4) Male and female bison from the Northern Range have different diet compositions, regardless of season. Regardless of time period or range, we expect females to have higher quality diets than males. Finally, during the mating season, males are most concerned with tending potential mates in an effort to maximize breeding potential, and thus, the variety of forages available to both males and females should be similar during this time. As a result, we expect males and females to have similar dietary breadths during the mating season, but statistically distinct dietary breadths throughout the multi-year period, regardless of range.

## Methods

### Study area

Yellowstone National Park occupies 891,000 ha of primarily forested habitat in northwestern Wyoming, USA, and ranges from 1500 to 3300 m in elevation [27]. During the summer months, the park is home to an estimated 3700 bison [29] that reside principally in two areas, the Central Range and the Northern Range (Fig. 1). As winter approaches, bison move to lower elevations, including the lower Yellowstone River drainage and Blacktail Deer Plateau in the Northern Range, where snow pack is shallower and the growth of spring vegetation begins earlier [30]. As noted above, distinct biotic and abiotic differences between the two ranges influence the abundance and distribution of high-quality forage for bison in YNP [23].

### Analytical methods

Fecal nitrogen is an established measure of dietary quality [31], and stable isotopes, especially of carbon and nitrogen, are commonly used to investigate dietary differences both within and among mammal populations [32]. The primary source of nitrogen in feces of mammalian herbivores is dietary protein, although plant secondary metabolites that bind to proteins as well as microbial sources of nitrogen may influence estimates of protein in feces [31]. Regardless, absolute forage intake [33], forage digestibility, and dietary protein are positively correlated with fecal N in grazing ungulates [34]. Stable isotopes of carbon found in the biogenic materials of herbivores (e.g., feces and collagen) reflect the average δ^13^C of plants ingested and assimilated during the formation of these materials [35, 36]. Thus, δ^13^C is commonly used to distinguish diet compositions of large herbivores using mathematical mixing models [37]. Even without the use of mixing models, however, both the mean and variance of stable isotopes provide information about dietary habits. Differences in mean δ^13^C have been used to analyze population differences in diet [38], and individual variation in δ^13^C from biogenic materials (e.g., feces and bone) provides a measure of dietary breadth within a population. As animals ingest a greater range of plant species and plant parts, the variance of δ^13^C increases [39, 40]. In general, the mean δ^15^N of animal tissues reflects the protein content of the animal’s diet [41, 42], with a negative correlation between the nitrogen content of ingested plants and δ^15^N values of herbivore bone collagen (δ^15^N_collagen_; [43]).

Stable isotopes of carbon and nitrogen from bone collagen and feces, along with fecal N content, provide information about diet over different temporal windows. Bone formation is a relatively continuous process [44], and bone collagen has a turnover rate on the order of years [45, 46]. Thus, the isotopic composition of bone collagen reflects the average foraging trends over much of the lifetime of sampled individuals (i.e., multi-year). Stable isotope values from bison fecal samples, in contrast, reflect the isotopic composition of forage ingested during the previous 24–48 h [47].

### Sample collection

We collected 60 fecal samples from bison in the Northern and Central ranges (15 males and 15 females from each range) over a period of 10 days during the mating season of early August 2009. We monitored animals along the periphery of the herd and when defecation occurred, we recorded a compass bearing in the direction of that potential sample, as well as any landmarks useful in determining the precise location of that sample (e.g., tree, bushes, flowers, mounds, etc.). No two samples were collected within close spatial proximity of one another (i.e., approximately 20 m) within the same herd, and samples were collected within 2 h of elimination. All fecal samples came from animals of reproductive age (≥6 years for males and ≥2 years for females); one fecal sample from the Central Range had to be discarded due to molding. We determined whether animals were of reproductive age based on size, head morphology, and pelage (mature males have well-defined manes that are absent on females and immature males; [48]).

The YNP scientific staff provided the locations of bison carcasses from adult animals that had died over the previous 36 months. Sample collection took place between 15 July and 7 August 2009. We collected 22 hemi-mandibles and 18 vertebrae from 40 carcasses. Twenty-five of the sampled carcasses were from the Central Range (13 females, 12 males), and 15 (11 females, 4 males) were from the Northern Range (Fig. 1). We estimated age at time of death using patterns of cheek–tooth eruption and wear [49] and the degree of fusion of suture lines on the frontal bones of the skull [50]. We determined the sex of carcasses based on sex-specific morphological differences in the skull [51] and pelvic girdle [52, 53]. For stable isotope analysis of bone collagen, we collected hemi-mandibles; when a hemi-mandible could not be located, we collected a single vertebra.

### Sample preparation and analysis

We dried fecal samples at 60 °C until stable weights were achieved, then homogenized them in a SPEX Certiprep 8000D ball mill. We weighed 0.3 ± 0.05 mg of each sample and wrapped it in a Costech 5 × 9 mm tin capsule for analysis of %N and δ^13^C. For stable isotope analysis of bone collagen, we demineralized approximately 50 mg of bone in 0.5 N HCl for 72 h at 4 °C. We then rinsed samples with de-ionized water and placed them in 6 ml of chloroform/methanol lipid-extraction solution [54]. Finally, we lyophilized the samples for 24 h and then wrapped 0.3 ± 0.05 mg of collagen sample in a Costech 5 × 9 mm tin capsule for analysis of δ^13^C and δ^15^N [55].

Samples were analyzed in the Nadelhoffer Stable Isotope Laboratory at the University of Michigan Biological Station (Pellston, Michigan). Fecal and collagen samples were analyzed on a Thermo Delta Plus XL isotope ratio mass spectrometer via combustion in a Costech CHN analyzer, Model 4010. Analytical precision was estimated via multiple analyses of a caffeine standard (mean δ^13^C = −49.35, SD = 0.16; mean δ^15^N = −1.7‰, SD = 0.12). Stable isotope values of C and N were reported to the nearest 0.01 and 0.1‰, respectively, and fecal-N content (%N_feces_) was reported to the nearest 0.01%. Data acquisition and instrument control were accomplished using ISODAT 2.0. Isotopic values of feces and bone collagen were reported relative to international standards, Vienna Pee Dee Belemnite (VPDB) for carbon and atmospheric N for nitrogen.

### Statistical analysis

We used multivariate analysis of variance (MANOVA) to determine whether diets differ throughout YNP as a function of sex and range. Specifically, we evaluated six different models (Table 1) to determine whether diets vary as a function of sex and range during the mating season (hypothesis 1, model 1) and over a multi-year period (hypothesis 2, model 2) and whether diets of males and females differ during the mating season and over a multi-year period in both the Central (hypothesis 3, models 3.1 and 3.2, respectively) and Northern ranges (hypotheses 4, models 4.1 and 4.2, respectively). For any tests resulting in *P* < 0.10, we applied a univariate ANOVA to determine whether any of the dependent variables were significantly influenced by our independent variables (i.e., sex and range).

**Table 1:** Structure and sample size for MANOVA models

H_a_	*x*	*y*	Time period	Sample size		Model ID
				Female	Male	
1	Sex	δ^13^C_feces_, %N_feces_	Mating season	30	29	1
2	Sex	δ^13^C_collagen_, δ^15^N_collagen_	Multi-year	24	16	2
3	Sex, CR	δ^13^C_feces_, %N_feces_	Mating season	15	14	3.1
		δ^13^C_collagen_, δ^15^N_collagen_	Multi-year	13	12	3.2
4	Sex, NR	δ^13^C_feces_, %N_feces_	Mating season	15	15	4.1
		δ^13^C_collagen_, δ^15^N_collagen_	Multi-year	11	4	4.2

Models tested whether diet varies as a function of sex and range between mating season and a multi-year time period. To test hypotheses 3 and 4, we subset the data for each range (CR, Central Range; NR, Northern Range), and analyzed the effect of sex on proxy measures for diet within each range. Columns labeled “*x*” and “*y*” denote the independent and dependent variables, respectively, for each model

**Fig. 2:**
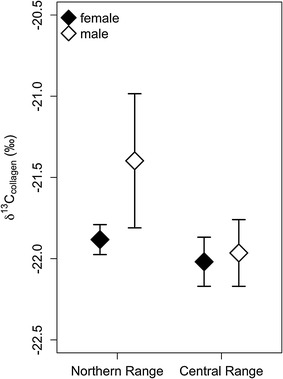
Mean and 95% confidence intervals for each combination of sex and range for δ^13^C of collagen (δ^13^C_collagen_). *Points* represent mean values for δ^13^C_collagen_ and *bars* represent 95% confidence intervals. Sample sizes for Northern Range bison are n = 11 for females and n = 4 for males, and sample sizes for Central Range bison are n = 13 for females and n = 12 for males

We also investigated differences in dietary breadth using Bartlett’s test for homogeneity of variance [56]. We compared variance of δ^13^C_feces_ to determine if dietary breadth varied as a function of sex and range during the mating season and compared variance of δ^13^C_collagen_ to determine whether dietary breadth varied as a function of sex across a multi-year time period. While our sample sizes may seem small when subdivided by sex and range, Clementz and Koch [57] reported that a sample size of *n* = *5* resulted in a standard error of 0.01‰ for the mean δ^13^C value of the study population and recommended ≥5 as suitable sample size for stable isotope studies of populations. However, one of our collagen test groups, Northern Range males, had a sample size just below this threshold. To determine the influence of this small sample size, we calculated 95% confidence intervals for each sex-range combination (Fig. 2). Additionally, we performed an F test to compare the variance of Northern Range males (n = 4) to Central Range males (n = 12) and found the variance between the two groups to be statistically indistinguishable (F = 1.3463, P = 0.6193). All statistical analyses were performed using base packages in R 3.1.2 [58].

## Results

**Fig. 3:**
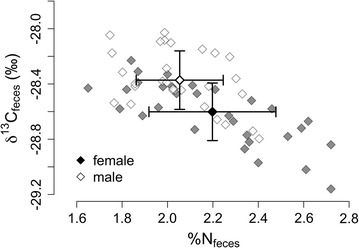
Stable isotope values of carbon (δ^13^C_feces_) and nitrogen content (%N_feces_) from bison feces. *Open symbols* represent males, and *filled symbols* represent females. *Bold diamonds with bars* represent mean ± 1 standard deviation, while *lighter diamonds* represent raw data

**Fig. 4:**
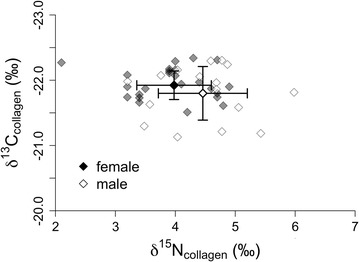
Stable isotope values of carbon (δ^13^C_collagen_) and nitrogen (δ^15^N_collagen_) from collagen tissue of male and female bison. *Open symbols* represent males, and *filled symbols* represent females. *Bold diamonds with bars* represent mean ± 1 standard deviation, while *lighter diamonds* represent raw data

Male and female bison throughout YNP had distinctly different diets during the mating season (Fig. 3; Table 2). While there was no difference in dietary breadth during this time (variance of δ^13^C_feces_, Table 2), mean values of δ^13^C_feces_ were significantly different between sexes indicating distinct diet compositions. Females had greater fecal nitrogen (%N_feces_) than males (Fig. 3; Table 3), indicating higher quality diets during the mating season. Throughout the multi-year period, there was no difference in mean δ^13^C_collagen_ (Fig. 4; Table 3) between sexes, indicating that males and females ingest similar plants or plant parts, on average. Despite having similar mean diet compositions, as a group, males had greater dietary breadth (variance of δ^13^C_collagen_, Table 2) during the multi-year period, whereas females had higher quality diets, as indicated by lower δ^15^N_collagen_ (Fig. 4; Table 3).

**Table 2:** Results for single-factor MANOVA tests and Bartlett’s tests for homogeneity of variance

Model ID	MANOVA		*P*	*K* ^*2*^	Bartlett’s test *P*
	df	*F*			
1	1,57	8.698	<0.001*	0.008	0.931
2	1,38	2.991	0.063	7.175	0.007*
3.1	1,27	11.915	<0.001*	1.718	0.190
3.2	1,23	1.434	0.260	0.781	0.377
4.1	1,28	2.025	0.153	1.430	0.232
4.2	1,13	5.403	0.021*	5.076	0.024*

Statistically significant results (*P* < 0.05) for both tests are identified with an asterisk. Bartlett’s test for homogeneity of variance was used to analyze differences in variance of δ^13^C_feces_ (mating season) and δ^13^C_collagen_ (multi-year)

**Table 3:** Univariate ANOVA results for MANOVA tests with *P* < 0.10

Model ID	*x*	*y*	Mean values		*df*	*SS*	*F*	*P*
			Female	Male				
1	Sex	δ^13^C_feces_	−28.61	−28.36	1,57	0.904	17.570	<0.001*
		% N_feces_	2.20	2.04	1,57	0.357	5.292	0.025*
2	Sex	δ^13^C_collagen_	−21.96	−21.82	1,38	0.171	1.536	0.223
		δ^15^N_collagen_	3.94	4.46	1,38	2.583	4.905	0.033*
3.1	Sex, CR	δ^13^C_feces_	−28.76	−28.38	1,27	1.056	18.670	<0.001*
		%N_feces_	2.46	2.14	1,27	0.713	20.410	<0.001*
4.2	Sex, NR	δ^13^C_collagen_	−21.88	−21.40	1,13	0.691	11.550	0.005*
		δ^15^N_collagen_	3.66	3.82	1,13	0.076	0.228	0.641

Significant difference in δ^13^C values between males and females indicates different dietary sources during each respective time period. Higher %N_feces_ indicates higher quality diets during the mating season, whereas lower δ^15^N_collagen_ indicates higher quality diet during the multi-year time period. Stable isotope values are reported to the nearest 0.01‰, and %N_feces_ is reported to the nearest 0.01%. Columns labeled “*x*” and “*y*” denote the independent and dependent variables, respectively, for each model

In the Central Range, male and female bison had significantly different diets during the mating season but not across the multi-year period (Table 2). Mean values of δ^13^C_feces_ differed significantly between sexes in the Central Range, indicating distinct diet compositions during the mating season. Females had greater %N_feces_ compared to males (Table 3), indicating higher quality diets; however, there was no difference in dietary breadth between sexes during this time (variance δ^13^C_feces_, Table 2). During the multi-year period, male and female diets in the Central Range were statistically indistinguishable in all regards, indicating diets of similar composition (δ^13^C_collagen_), quality (δ^15^N_collagen_), and breadth (variance δ^13^C_collagen_, Table 2).

In contrast to what we observed in the Central Range, male and female bison in the Northern Range had significantly different diets during the multi-year period but not during the mating season (Table 2). During mating season, male and female diets in the Northern Range were statistically indistinguishable in all regards, indicating diets of similar composition, quality, and breadth (Table 2). However, during the multi-year period, δ^13^C_collagen_ differed significantly between sexes, indicating sex-specific differences in mean diet composition (Table 3). Finally, although males had greater dietary breadth (variance δ^13^C_collagen_, Table 2) during the multi-year period in the Northern Range, there was no difference in diet quality (δ^15^N_collagen_, Table 3).

## Discussion

We studied whether diet segregation of bison in YNP is associated with sex-specific nutritional demands and whether these demands are met differently in each range. We tested four hypotheses and found complete or partial support for each. Specifically, we found that mean diet composition of male and female bison during the mating season differs significantly, with females having higher quality diets and males having greater dietary breadth (hypothesis 1). Further, while mean diet composition for male and female bison throughout multiple years is statistically indistinguishable, females have higher quality diets and males have greater dietary breadth (hypothesis 2). Additionally, diet segregation for bison in the Central Range was more pronounced during the mating season than across the multi-year period; while females had higher quality diets than males during this time, there was no difference in dietary breadth (hypothesis 3). Finally, diet segregation in the Northern Range was more pronounced across the multi-year period than during the mating season; while males had greater dietary breadth during this time, there was no difference in diet quality (hypothesis 4).

Our results suggest that diet segregation of bison in YNP is associated with sex-specific nutritional demands and density-dependent influences associated with meeting these demands. During mating season, the diets of male and female bison are composed of different plants or plant parts (based on differences in δ^13^C_feces_), with females consuming higher quality diets on average (higher %N_feces_). Although the difference in δ^13^C_feces_ between sexes is small (0.25‰), it represents more than 20% of the total range of δ^13^C_feces_ values. Over the multi-year time period, male and female bison, on average, consume a similar array of plants or plant parts throughout the park (based on similarity of δ^13^C_collagen_). Despite the similarity in average diet composition, however, females have higher quality diets (lower δ^15^N_collagen_) than males and appear to be more consistent in their use of available forages (lower variance in δ^13^C_collagen_). Collectively, these results suggest that females exhibit more selective feeding behavior throughout the majority of the year compared to males. Numerous field studies of ungulates have reported that, while different sexes may forage in the same habitats, their diets can differ significantly, with females usually selecting forage of higher quality [7, 59, 60]. For example, dietary overlap of male and female white-tailed deer decreases during periods of aggregation (e.g., mating season) compared to periods of segregation [61], and fecal nitrogen of females is greater than that of males, regardless of season [62].

Despite the fact that the two bison populations studied are separated by only tens of kilometers, we found evidence of opposing responses of sex-specific diet segregation in the two ranges. In the Central Range, diet segregation in bison was apparent during the mating season but not during the multi-year period, whereas in the Northern Range, diet segregation was apparent during the multi-year period but not during the mating season. In the Central Range, although males and females obtained a majority of their forage from different plants or plant parts during the mating season, with females ingesting higher quality forage, there was no difference in dietary breadth. Over the multi-year period, males and females from the Northern Range obtained a majority of their forage from different plants or different plant parts. While there was no sex-specific difference in dietary quality for Northern Range bison during this time, males consumed a greater diversity of dietary items compared to females. Opposing responses of diet segregation in each range may result from differences in the abundance and distribution of high-quality forage and the varying degree of competition for this forage across different ranges and different time periods.

While the Yellowstone ecosystem is considered to be forage-limited for its ungulate herbivores, competition for forage is likely greater on the Northern Range than on the Central Range [26]. The Northern Range offers high-quality forage throughout much of the year, and population densities of elk and bison are greater on the Northern Range, especially during the mating season [27, 28]. Although competition for forage peaks during the mating season for bison in both the Northern and Central ranges, range-specific differences in the availability of high-quality forage may help explain the differences in diet segregation.

Another important consideration is the abundance and distribution of potential predators. While predation rates on bison vary throughout the park, elk are the primary prey of wolves in Yellowstone. In the Northern Range, where elk are most abundant, bison make up only about 4% of wolf kills, with elk comprising approximately 80% of kills [21]. Elk are much less abundant in the Central Range, and as a result, wolf predation rates on bison are higher there than anywhere else in the park, with approximately 10% of wolf kills in this region consisting of bison and around 40% of kills consisting of elk [20]. Moreover, while bison populations without predators have been characterized by the forage selection hypothesis [5, 16], populations with predators have been characterized by the predation risk hypothesis [60], in which females select habitats that offer increased safety at the cost of decreased food quality [63, 64]. Evidence suggests that the presence of potential predators can result in decreased foraging activity [65, 66], thereby directly influencing diet quality of prey [67]. Altogether, our results suggest that bison in Yellowstone National Park are best characterized by the forage selection hypothesis.

Although we interpret our results in the context of the forage selection hypothesis, our results are also consistent with the niche trade-off framework described by Bowyer [8], in which overlap on one niche axis (e.g., space) is accompanied by avoidance on another (e.g., diet). During mating season in YNP, females outnumber males by about 2–1 [68, 69]. Thus, males may be at a competitive disadvantage for those plants that females prefer but that make up at least a portion of male diets during the rest of the year. However, intraspecific competition as a driver of diet segregation in ruminants has been rejected several times in the literature [70, 71], and testing this hypothesis is beyond the scope of this study. Although higher densities of females during the mating season may exclude males from consuming certain forages, these differences are best explained by morphological differences between sexes resulting in different physiologies and therefore different nutritional demands. Specifically, it has been hypothesized that a significant increase in the ingestion of high-quality forage by males could lead to mal-absorption, bloat, and ultimately, decreased digestive efficiency [72]. For comparison, in southern Texas, adult white-tailed deer shifted to lower quality diets as levels of intraspecific competition increased, with dietary shifts of males being more pronounced than those of females [61]. However, the authors rejected competition as a cause for sexual segregation and suggested that predator avoidance by females with young best explained the observed pattern [61]. Similarly, bighorn sheep in Sierra Nevada, California exhibited stronger niche differentiation during periods of spatial overlap than during periods of spatial separation [73]; however, the authors noted that these differences are most likely explained either by morphological differences or by how different sexes manage risk of predation [73]. It is also important to note that male ruminants tend to restrict feeding behavior during mating season [74], potentially resulting in decreased dietary breadth and lower variance of δ^13^C during periods of aggregation.

Finally, many studies investigating isotopic variation among conspecifics on different dietary regimes or in different regions have found only minor differences in isotope values between test groups. For example, several populations of mule deer with significant differences in fecal N content exhibited only small differences in mean δ^15^N_feces_, ranging from −0.54 to 1.10‰ [75]. Similarly, a study investigating the isotopic composition of hair in cattle (*Bos taurus*) from different confinement and dietary practices found that six of 13 test groups had mean δ^13^C values within a range of less than 1‰ [76]. Cattle, elk, and mule deer in Starkey Experimental Forest, Oregon, had a collective δ^13^C range of less than 2‰ and a collective δ^15^N range of less than 4‰ [39]. In YNP, Feranec reported that mean δ^13^C values of feces collected over a 1-month period from bison, elk, mule deer, and white-tailed deer (*O*. *virginianus*) fell within a range of less than 1‰, and mean δ^13^C values of collagen for bison, elk, and mule deer lay within a range of about 2‰ [77]. Moreover, of the large herbivores resident to YNP, bison had the smallest δ^13^C range for feces and collagen, both of which were 0.9‰. Thus, while differences in forage type, feeding strategy (e.g., browser vs. grazer), and ecological setting may result in only small differences in isotopic values, these differences may correspond to biologically significant variations in diet [78]. Finally, while some of our sample sizes may seem small for groups divided by sex and range, all but one sample size was well over the recommended threshold for stable-isotopic characterization of populations [53]. For the sample that lay below this threshold, Northern Range males, the variance is statistically indistinguishable from that of Central Range males, which has three times the number of samples. Regardless, this particular result should be considered preliminary. Also, the threshold value for sample size was determined for enamel carbonate samples, not for collagen and fecal isotopes; however, more recent studies corroborate these findings using collagen isotopes [79].

## Conclusions

Ecological theory suggests that smaller-bodied, female bison should displace males from high-quality foraging habitats, since females forage in large groups and deplete resources more rapidly than do males [2, 3, 7]. Based on our results, female bison in YNP have higher quality diets than males do, suggesting that diet segregation is associated with sex-specific nutritional demands. Additionally, range-specific differences in the abundance and distribution of high-quality forage, in conjunction with seasonal variation in population density of bison and elk, may influence spatial and temporal differences in diet segregation. Altogether, our results highlight the importance of accounting for spatiotemporal heterogeneity when conducting dietary studies on wild ungulates.
